# Evaluation of Anti-CAR Linker mAbs for CAR T Monitoring after BiTEs/bsAbs and CAR T-Cell Pretreatment

**DOI:** 10.3390/biomedicines12081641

**Published:** 2024-07-24

**Authors:** Anja Grahnert, Sabine Seiffert, Kerstin Wenk, Dominik Schmiedel, Andreas Boldt, Vladan Vucinic, Maximilian Merz, Uwe Platzbecker, Christian Klemann, Ulrike Koehl, Maik Friedrich

**Affiliations:** 1 Medical Faculty, Institute of Clinical Immunology, Leipzig and University of Leipzig Medical Center, Leipzig University, 04103 Leipzig, Germany; anja.grahnert@medizin.uni-leipzig.de (A.G.); sabine.seiffert@medizin.uni-leipzig.de (S.S.); kerstin.wenk@medizin.uni-leipzig.de (K.W.); andreas.boldt@medizin.uni-leipzig.de (A.B.); ulrike.koehl@izi.fraunhofer.de (U.K.); 2 Fraunhofer Institute for Cell Therapy and Immunology, 04103 Leipzig, Germany; dominik.schmiedel@izi.fraunhofer.de; 3 Department of Hematology and Cell Therapy, Medical Faculty, Leipzig and University of Leipzig Medical Center, Leipzig University, 04103 Leipzig, Germany; vladan.vucinic@medizin.uni-leipzig.de (V.V.); maximilian.merz@medizin.uni-leipzig.de (M.M.); uwe.platzbecker@medizin.uni-leipzig.de (U.P.); 4 University Cancer Center Leipzig (UCCL), Cancer Center Central Germany (CCCG), 04103 Leipzig, Germany; 5 Department of Pediatric Immunology, Pediatric Rheumatology and Infectiology, Clinic and Polyclinic for Pediatric and Adolescent Medicine, Leipzig and University of Leipzig Medical Center, Leipzig University, 04103 Leipzig, Germany; christian.klemann@medizin.uni-leipzig.de

**Keywords:** anti-CAR-linker mAbs, CAR T-cell monitoring, bispecific antibody, BiTE, combination treatments, diagnostic, CAR, detection

## Abstract

For the monitoring of chimeric antigen receptor (CAR) T-cell therapies, antigen-based CAR detection methods are usually applied. However, for each target-antigen, a separate detection system is required. Furthermore, when monitored CAR T-cells in the blood of patients treated with bispecific antibodies or T-cell engagers (bsAbs/BiTEs) recognize the same antigen, these methods produce false-positive results in clinical diagnostics. Anti-CAR-linker monoclonal antibodies (mAbs) targeting the linker sequence between the variable domains of the antigen binding CAR fragment promise a universal and unbiased CAR detection. To test this, we analyzed clinical specimens of all BCMA- and CD19-targeting CAR T-cell products currently approved for clinical use. We found a highly specific and sensitive CAR detection using anti-CAR-linker mAb in blood cells from patients treated with Ide-cel, Tisa-cel, Axi-cel, Brexu-cel, and Liso-cel. For Ide-cel and Tisa-cel, the sensitivity was significantly lower compared to that for antigen-based CAR detection assays. Strikingly, the specificity of anti-CAR linker mAb was not affected by the simultaneous presence of bispecific blinatumomab or teclistamab for Axi-cel, Brexu-cel, Liso-cel, or Ide-cel, respectively. Cilta-cel (containing a monomeric G_4_S-CAR linker) could not be detected by anti-CAR linker mAb. In conclusion, anti-CAR-linker mAbs are highly specific and useful for CAR T-cell monitoring but are not universally applicable.

## 1. Introduction

To treat hematologic malignancies, chimeric antigen receptor (CAR) T-cells are increasingly used due to their safety and high efficacy [1,2,3]. Clinically approved CAR therapies are directed against B cell malignancies such as multiple myeloma (MM), acute lymphocytic leukemia, and lymphomas, are resistant to conventional treatments [4,5,6,7]. These second-generation CARs consist of an antibody-derived extracellular ligand-binding domain, linked to a spacer and transmembrane domain (CD8a, CD28, or IgG) and two cytoplasmic domains providing co-stimulatory and activating signals to the T-cell (4-1BB/CD3ζ or CD28/CD3ζ, respectively) [8]. The ligand-binding domains are mostly engineered as single-chain variable fragments (scFv) able to bind to a tumor antigen. The CAR redirects the T-cell to identify and kill the antigen-carrying tumor cell. In our cancer center we applied all CAR T-cell therapies currently approved for clinical use in Europe: tisagenlecleucel (Tisa-cel; Kymriah^®^, Novartis, Basel, Switzerland), axicabtagene ciloleucel (Axi-cel; Yescarta^®^, Kite Pharma, Santa Monica, California, USA), brexucabtagene autoleucel (Brexu-cel; Tecartus^®^, Kite Pharma, Santa Monica, California, USA), and lisocabtagene maraleucel (Liso-cel; Breyanzi^®^, Bristol Myers Squibb, New York City, USA), idecabtagene vicleucel (Ide-cel; Abecma^®^, Celgene Corporation, Summit, New Jersey, USA), and ciltacabtagene autoleucel (Cilta-cel; Carvykti^®^, Janssen-Cilag, North Ryde, New South Wales, Australia) [9,10]. These CAR T-cell products used to treat B-cell leukemias and lymphomas (Tisa-cel, Axi-cel, Brexu-cel, and Liso-cel) or MM (Ide-cel and Cilta-cel) bind to the extracellular domain of the CD19 antigen or the B cell maturation antigen (BCMA), respectively.

The manufacturing process of autologous CAR T-cell products includes lymphocyte apheresis, lentiviral transfection of the CAR into the T-cells, followed by cell expansion and subsequent infusion into the patient [11]. Monitoring the course of CAR T-cell treatment numbers and frequencies of CAR T-cells in individual patients is pivotal for diagnosis [1,12,13]. Therefore, antigen-based flow cytometric CAR T-cell detection methods are feasible for clinical routine diagnostics [12,14]. These assays recognize the binding of a biotin-labeled recombinant antigen to the antigen-binding site of the CAR, detected by a fluorescent labeled anti-biotin antibody [12,15].

In addition to CAR T-cells, another therapeutic strategy that redirects the patient’s T-cell cytotoxic activity to cancer cells consists of artificial bispecific antibodies (bispecific T-cell engagers, BiTEs) and bispecific antibodies (bsAbs) [16,17,18]. 

One arm of a BiTE/bsAb binds to a surface antigen of a tumor cell (e.g., CD19 or BCMA), whereas the other arm binds to the CD3 receptor of a T-cell initiating a T-cell receptor-independent killing of cancer cells. As the combination of BiTE/bsAb and CAR T-cells has been demonstrated to improve the effectiveness of CAR T-cell therapy, either as bridging or as combined therapeutic approaches, both are now increasingly used in practice or in clinical trials [19], respectively.

Previously, we have demonstrated that teclistamab, a BCMA×CD3 bsAb [20], interferes with flow cytometry-based BCMA CAR T-cell detection in the blood of MM patients, thus leading to false positive results [12]. Teclistamab binds to all T-cells via CD3, irrespective of the presence of a CAR, while the other arm (even in the absence of a CAR) binds the soluble biotinylated BCMA used for CAR detection. More generally, CAR monitoring in the presence of BiTEs/bsAbs is not possible for CARs/BiTEs/bsAbs combinations recognizing the same antigen (e.g., anti-CD19 CARs/CD19×CD3 BiTEs), if antigen-based CAR detection methods are applied [12].

To circumvent this critical diagnostic problem, we here evaluate a new detection strategy using anti-CAR linker monoclonal antibodies (mAbs) [13,21,22], targeting the linker sequence between the variable heavy and variable light domains of the scFv.

## 2. Materials and Methods

### 2.1. Reagents and Antibodies

Biotin-labeled BCMA CAR detection reagent, biotin-labeled CD19 CAR detection reagent, and anti-biotin APC were obtained from Miltenyi Biotec (Bergisch-Gladbach, Germany). Anti-G_4_S CAR linker rabbit mAb (E7O2V), anti-Whitlow CAR linker rabbit mAb (E3U7Q) (biotinylated or Alexa Fluor^®^ 647-conjugated), and rabbit IgG XP^®^ isotype-Alexa Fluor^®^ 647 (DA1E) were obtained from Cell Signaling Technology Europe B.V. (Leiden, The Netherlands). Anti CD3-FITC Ab (SK7), anti CD45-V500C Ab (2D1), BD FACS™ lysing solution, and BD Pharm Lyse™ lysing buffer were obtained from Becton Dickinson (Heidelberg, Germany). Anti-CD3 Ab and anti-CD28 Ab for T-cell stimulation are from eBioScience (distributed via Thermo Fisher Scientific, Waltham, MA, USA).

### 2.2. Patient Samples

EDTA- or Heparin-anticoagulated blood was obtained from patients suffering from B-cell malignancies (MM, leukemia, or lymphoma) at day 7–23 after application of CAR T-cell therapy with idecabtagene vicleucel (Ide-cel, n = 5), ciltacabtagene autoleucel (Cilta-cel, n = 5), tisagenlecleucel (Tisa-cel, n = 4), axicabtagene ciloleucel (Axi-cel, n = 4), brexucabtagene autoleucel (Brexu-cel, n = 3), or lisocabtagene maraleucel (Liso-cel, n = 2). Ethical approval was granted by the ethics committee of Leipzig University (429/21-lk).

### 2.3. Separation of PBMC from Whole Blood and T-Cell Subculture 

Human peripheral blood mononuclear cells (PBMCs) from patients’ EDTA- or Heparin-anticoagulated blood were obtained by Lymphoprep™ (Progen Biotechnik GmbH, Heidelberg, Germany) density centrifugation. After repeated washing with cold PBS (Thermo Fisher Scientific, Waltham, MA, USA), 1 × 10^6^ PBMCs were cultured overnight in 1 mL X-VIVO 10™ media (Lonza, Walkersville, MD, USA) supplemented with 2% heat inactivated human AB-sera (Merck KGaA, Darmstadt, Germany) and human IL2 (200 U/mL, Peprotech, Hamburg, Germany).

To activate the isolated T-lymphocytes, cells were transferred to a culture plate precoated with anti-human CD3 (5 µg/mL) and costimulated with soluble anti-CD28 Ab (2 µg/mL). After 2–3 days, cells were transferred to X-VIVO 10™ media (supplemented with human AB-sera and human IL2) without stimulus. All cell cultures steps were performed at 37 °C, 5% CO_2_.

### 2.4. Bispecific Antibody Treatment

Blood samples from CAR T-cell treated patients (1 mL) or T-cells subcultured from patients’ blood (1 × 10^6^ cells in 1 mL PBS) were incubated with or without teclistamab (10 µg/mL) for 10 min at RT. Cells were washed twice with 1 mL PBS containing 2% heat-inactivated fetal calf serum (FCS; Gibco distributed via Thermo Fisher Scientific, Waltham, MA, USA) and analyzed by flow cytometry.

### 2.5. Antigen-Based CAR T-Cell Detection by Flow Cytometry

Biotin-labeled BCMA CAR detection reagent (2 µL) or biotin-labeled CD19 CAR detection reagent (2 µL) was added to EDTA-anticoagulated blood (100 µL) or to T-cell subcultures from patients’ blood (2 × 10^5^ cells in 100 µL PBS) and incubated for 15 min at room temperature (RT). After the addition of PBS and centrifugation (400 g, 5 min, RT), cells were resuspended in 100 µL PBS and incubated with 2 µL anti-biotin-APC Ab, 5 µL anti CD3-FITC Ab, and 2.5 µL anti CD45-V500C Ab for 15 min at RT. 

In assays using blood specimens, 2 mL of BD FACS™ lysing solution (Becton Dickinson, Heidelberg, Germany) or 2 mL BD Pharm Lyse™ lysing buffer (Becton Dickinson, Heidelberg, Germany) was added for 10 min or 15 min, respectively. After washing and fixation, the cells were analyzed using a FACSLyric flow cytometer (Becton Dickinson). BD FACSuite™ software 1.6 (Becton Dickinson) was used for data analysis.

Cultured T-cells were subsequently washed twice and resuspended in 100 µL PBS with 5 µL of 7-AAD. After 10 min at RT, the cells were analyzed using a FACSLyric flow cytometer (Becton Dickinson). BD FACSuite™ software 1.6 (Becton Dickinson) was used for data analysis. 

### 2.6. CAR Linker-Based CAR T-Cell Detection by Flow Cytometry

Biotin-labeled anti-G_4_S CAR linker mAb (4 µL), biotin-labeled anti-Whitlow CAR linker mAb (4 µL), anti-G_4_S CAR linker-Alexa Fluor^®^ 647 mAb (4 µL), anti-Whitlow CAR linker-Alexa Fluor^®^ 647 mAb (4 µL), or rabbit IgG XP^®^ isotype-Alexa Fluor^®^ 647 were added to EDTA-anticoagulated blood (100 µL) or to T-cell subcultures from patients’ blood (2 × 10^5^ cells in 100 µL PBS) and incubated for 15 min at RT. After the addition of PBS and centrifugation (400 g, 5 min, RT), cells were resuspended in 100 µL PBS and incubated with 5 µL anti CD3-FITC Ab, 2.5 µL anti CD45-V500C Ab, and 2 µL anti-biotin antibody APC (in case of the biotin-labeled Abs) for 15 min at RT.

In assays using blood specimens, 2 mL of BD FACS™ lysing solution (Becton Dickinson, Heidelberg, Germany) or 2 mL BD Pharm Lyse™ lysing buffer (Becton Dickinson, Heidelberg, Germany) was added for 10 min or 15 min, respectively. After washing and fixation, the cells were analyzed using a FACSLyric flow cytometer (Becton Dickinson). BD FACSuite™ software 1.6 (Becton Dickinson) was used for data analysis.

Cultured T-cells were subsequently washed twice and resuspended in 100 µL PBS with 5 µL of 7-AAD. After 10 min at RT, the cells were analyzed using a FACSLyric flow cytometer (Becton Dickinson). BD FACSuite™ software 1.6 (Becton Dickinson) was used for data analysis.

### 2.7. Statistical Analyses

All statistical analyses were performed using SigmaPlot software 14.0 (Systat Software, Erkrath, Germany). Statistical significance was calculated with Student’s *t*-test and classified as follows: * *p* ≤ 0.05; ** *p* ≤ 0.01; *** *p* ≤ 0.001.

## 3. Results

**Figure 1:**
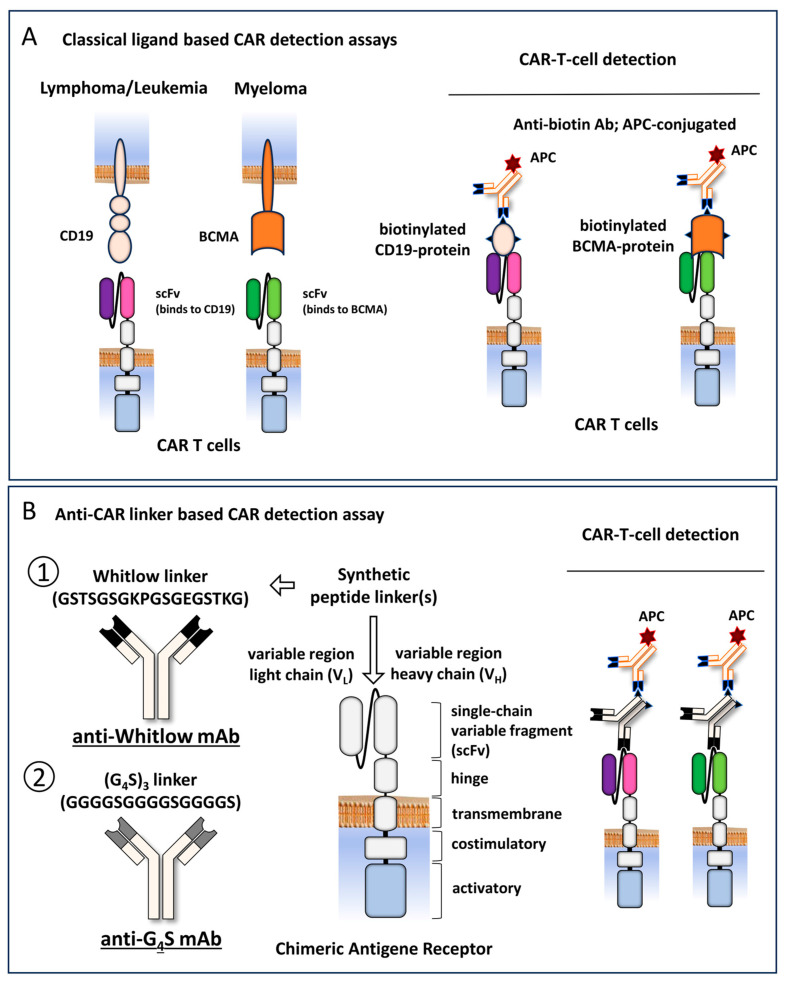
Schematical representation of CAR T-cell detection with the classical ligand-based assay and anti-CAR linker mAb. (**A**) A schematical representation of CD19 and BCMA tumor antigens and their targeting CARs are shown. The recombinantly produced and biotinylated ligands and APC-conjugated anti-biotin Abs are used to visualize CAR expression on the surface of T-cells. (**B**) The general structure of a chimeric antigen receptor (CAR) is depicted as an illustration. The single-chain variable fragment (scFv) of CARs contains either a Whitlow linker (GSTSGSGKPGSGEGSTKG) or a (G_4_S)_3_ linker (GGGGSGGGGSGGGGS), targeted by anti-Whitlow mAb or by anti-G_4_S monoclonal antibody (mAb), respectively. Biotinylated CAR-linker mABs and APC-conjugated anti-biotin Abs are used for the universal visualization of CAR expression on the surface of T-cells.

**Figure 2:**
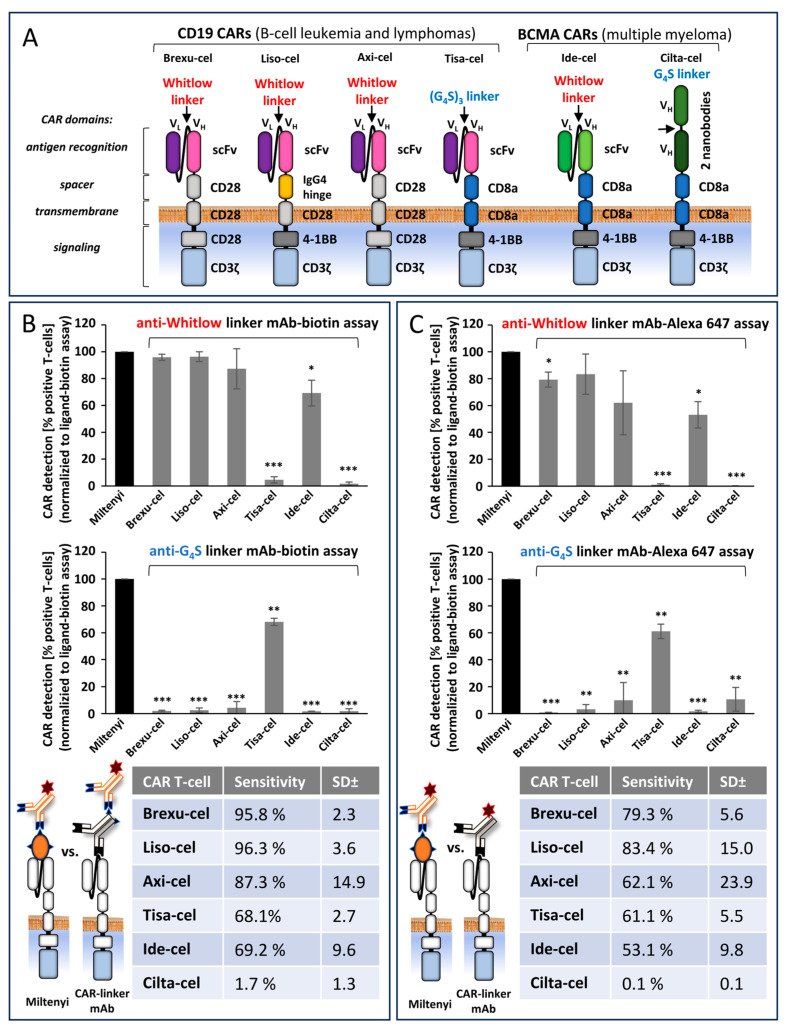
Anti-CAR linker mAbs allows for specific and sensitive CAR T monitoring in patients treated with any clinically approved CAR T therapies except Cilta-cel. (**A**) The structures of BCMA- and CD19-specific CARs are depicted in the illustration. CD19 antigen-specific CARs: Brexu-cel, Liso-cel, Axi-cel, and Tisa-cel; BCMA-specific CARs: Ide-cel and Cilta-cel. (**B**) Flow cytometry-based CAR detection analyses are shown, performed with biotinylated anti-CAR linker mAbs and APC-conjugated anti-biotin Abs to all approved BCMA and CD19 CAR T-cell products using isolated lymphocyte specimens from patients’ blood (n = 3). (**C**) Flow cytometry-based CAR detection analyses are shown, performed with Alexa 647-conjugated anti-CAR linker mAbs to all approved BCMA and CD19 CAR T-cell products using isolated lymphocyte specimens from patients’ blood (n = 3). The CAR-positive cells were determined from the CD3-positive cells and significances calculated based on BCMA- and CD19-antigen-based CAR detection reagent (Miltenyi = 100%). Statistical analysis was performed using Student’s *t*-test. * *p* ≤ 0.05%; ** *p* ≤ 0.01%; *** *p* ≤ 0.001%.

Antigen-based flow cytometric CAR T-cell detection methods are most frequently used for clinical routine diagnostics. These methods are based on the binding of a biotin-labeled recombinant ligand to the antigen-binding site of the CAR, detected by a fluorescent labeled anti-biotin antibody. For each CAR recognizing a different target-antigen, a separate biotin-labeled recombinant antigen is required (Figure 1A). Most scFv-based CARs contain either a Whitlow linker (GSTSGSGKPGSGEGSTKG) or a (G_4_S)_3_ linker (GGGGSGGGGSGGGGS), targeted by anti-Whitlow mAb or by anti-G_4_S mAb, respectively [13,21,22] (Figure 1B). To test whether these antibodies allow for a universal CAR detection in real life, we performed flow cytometry CAR detection analyses of all approved CD19- and BCMA-targeting CAR T-cell products, using isolated lymphocyte specimens from patients’ blood (Figure 2A). 

The percentage of anti-CD19 CAR T-cells detected by biotinylated anti-Whitlow mAb from lymphoma patients treated with Brexu-cel, Liso-cel, and Axi-cel was comparable to that obtained with CD19 antigen-based CAR detection assays (96 ± 2%; 96 ± 4%; and 87 ± 15%; respectively) (Figure 2B). Using biotinylated anti-G_4_S mAb, the percentage of Tisa-cel CAR T-cells detected in blood cell isolates from treated leukemia patients, only amounted to 68 ± 3% (Figure 2B). The use of fluorochrome-conjugated anti-CAR linker mAbs resulted in a reduced sensitivity of 79 ± 6%, 83 ± 15%, 62 ± 24%, and 61 ± 6% for Brexu-cel, Liso-cel, Axi-cel, and Tisa-cel, respectively (Figure 2C). These results indicate that detection with an anti-biotin secondary antibody amplifies the signal with respect to fluorochrome-conjugated anti-linker mAbs.

Furthermore, we performed flow cytometry CAR detection analyses of all approved BCMA CAR T-cell products (Ide-cel and Cilta-cel) (Figure 2A–C). As result, anti-BCMA CAR T-cells were also successfully detected by anti-Whitlow mAb from myeloma patients treated with Ide-cel. In comparison to the results obtained with BCMA antigen-based CAR detection assays, the percentage of detected CAR T-cells was reduced (69 ± 10% and 53 ± 10% for biotinylated and Alexa 647-conjugated anti-Whitlow mAb, respectively). However, the detection of Cilta-cel (featuring two single domain antibodies, connected by a single G_4_S linker) was not possible by any CAR linker mAb.

From a technical perspective, CAR detection with Alexa-conjugated anti-G_4_S mAb leads to false positive results in whole blood specimens, if red blood cells are lysed by BD FACS™ Lysing solution (Figure S1). This phenomenon occurs in all analyzed cells, but not with the corresponding Alexa-conjugated isotype, anti-CD3 Ab and anti-CD45 Ab. We speculate that an artificial binding of that antibody by stickiness may have occurred through simultaneous fixation by the lysing buffer in the diagnostic routine protocol. This artifact can be avoided using BD Pharm Lyse™ lysing solution, devoid of fixation reagent (Figure S1).

**Figure 3:**
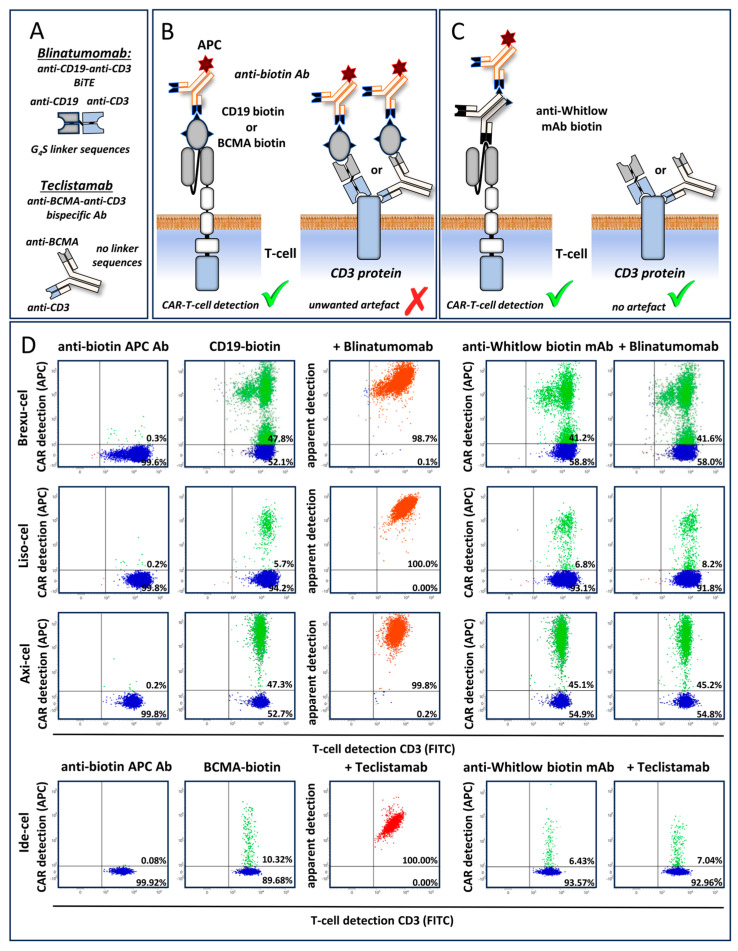
Anti-CAR linker mAbs allows for specific CAR T monitoring in patients pretreated with BiTE/bsAb, targeting the same antigen as the CAR. (**A**) The structure of the blinatumomab (CD19×CD3 BiTE) and teclistamab (BCMA×CD3 bsAb) are illustrated. (**B**) Shown is a schematic overview of CAR T-cell detection strategies based on the CD19 or BCMA CAR detection reagent (Miltenyi) that leads to a false positive staining in patients pretreated with BiTE/bsAb targeting the same antigen as the CAR. The structure of CD19- or BCMA-specific CAR and CD3 on the cell surface of a T-cell is depicted as an illustration. (**C**) Shown is a schematic overview of CAR T-cell detection strategies based on anti-CAR linker mAbs. The specificity of the CAR detection is not affected by BiTE/bsAb targeting the same antigen as the CAR. (**D**) Detection of CAR-positive cells in CD3-positive blood T-lymphocytes from patients, treated either with Brexu-cel, Liso-cel, and Axi-cel (anti-CD19 CAR T-cells) simultaneously in the presence or absence of blinatumomab BiTE (CD19×CD3) or treated either with Ide-cel (anti-BCMA CAR T-cells) simultaneously in the presence or absence of teclistamab bsAb (BCMA×CD3). Representative clinical blood specimens of a Lymphoma or MM patient are shown as dot plots. CAR-negative and CAR-positive cells are visualized using blue and green dots, respectively. Green and red checkmarks denote for specific or artificial CAR T-cell detection, respectively. Shown are the CD3-positive cells. Note that CD19- and BCMA-based CAR detection reagents (Miltenyi) interfere with blinatumomab and teclistamab, respectively, and bind to CD3 on the surface of all T-cells, irrespective of whether they carry a CAR or not. This leads to false positive results (denoted by dots shown in orange or red). The problem is solved using anti-CAR linker mAbs targeting the artificial linker sequence between the variable heavy- and light-chain domains of the scFv.

We next determined whether CAR linker mAb allows for the specific detection of CAR T-cells by the simultaneous presence of bsAbs/BiTE recognizing the same tumor antigen (e.g., CD19×CD3 BiTE blinatumomab and anti-CD19 CAR T-cells) (Figure 3A–C). 

Here, we show that the detection of anti-CD19 CAR T-cells with biotinylated anti-Whitlow mAbs in blood specimens of lymphoma patients treated with Brexu-cel is unaffected by the presence or absence of blinatumomab (41% versus 42%, respectively), in contrast to the CD19-based CAR detection assay (artificially 99% versus 48%, respectively) (Figure 3D). Similar results were obtained using Liso-cel and Axi-cel anti-CD19 CAR T-cells and blinatumomab (Figure 3D). Furthermore, the detection of BCMA specific CAR T-cells after prior treatment with BCMA×CD3 bsAb teclistamab is also impaired if BCMA antigen-based CAR detection reagents are used (Figure 3A-C). We show that Ide-cel (anti-BCMA) CAR T-cell detection by biotinylated anti-Whitlow Abs is also unaffected in the presence or absence of teclistamab (7% versus 6%, respectively), in contrast to the BCMA-based CAR detection assay (artificially 100% versus 10%, respectively) (Figure 3D).

Finally, the real-world feasibility for anti-CAR linker antibodies is identical to classical CAR detection, but the costs are considerably lower. In our laboratory, the costs per application are € 40.80 and € 30.90 for the classical CD19- and BCMA-based CAR detection assays, respectively, in contrast to € 23.00 and € 21.83 for the anti-Whitlow and anti-G_4_S linker mAb-based CAR detection assays, respectively. 

## 4. Discussion

In the domain of cellular immunotherapy, the concurrent administration of bispecific T-cell engagers (BiTEs) or bispecific antibodies (bsAbs) and chimeric antigen receptor (CAR) T-cell therapies targeting the same antigen presents a significant challenge for the accurate monitoring of CAR T-cell populations. The monitoring of a CAR T-cell therapy in cases where BiTEs/bsAbs recognizes the same antigen is not possible by antigen-based CAR detection methods. The BiTEs/bsAbs bind to CD3 on all T-cells irrespective of whether they carry a CAR or not. In this case, antigen-based CAR detection does not only bind to the CAR but also to the antigen-specific free arm of the bound BiTEs/bsAbs. This non-selective binding results in the misidentification of non-CAR-bearing T-cells as CAR-positive, thereby producing false-positive results in clinical therapy monitoring. 

For clinical diagnostics, this is a serious problem, as a combination of BiTE/bsAbs and CAR T-cells is employed either as a bridging therapy or as a combined therapeutic approach in clinical practice or clinical trials, respectively [19]. We still observed in our clinical routine, diagnostic false-positive results on day 9 after the last teclistamab treatment. 

The removal of the BiTEs/bsAbs from the cells before detecting the CAR might solve that problem. However, the in vitro application of reducing agent dithiothreitol [23] failed to completely disrupt teclistamab binding during CAR detection [12]. Alternative CAR-detection strategies based on Protein L, polyclonal anti-F(ab) fragment Abs (Figure S2), and anti-isotype antibodies are less specific [24], and would also label BiTEs/bsAbs bound to all T-cells in most cases. In addition, anti-idiotype antibodies are only available for the detection of a limited number of CARs [12,24].

Here, for the first-time to our knowledge, we evaluated anti-CAR-linker-specific monoclonal antibodies (mAbs) as a CAR detection strategy to circumvent the artificial staining of T-cells in the presence of corresponding BiTEs/bsAbs. These mAb target the linker sequence between the variable heavy and variable light domains of the scFv [13,21,22]. The scFv of the clinically applied CARs mostly contain a Whitlow protein linker (Ide-cel, Axi-cel, Brexu-cel, and Liso-cel) and were specifically detected by anti-Whitlow mAb. Tisa-cel is the only one CAR product, containing a (G_4_S)_3_ linker and was specifically detected by anti-G_4_S mAb. Surprisingly, the detection of Cilta-cel is not possible with anti-CAR linker antibodies. Cilta-cel uses two nanobodies (single domain antibody, naturally found in sharks and camels) instead of an scFv to recognize two epitopes of BCMA [25,26]. Both nanobodies are connected by a G_4_S linker instead of a (G_4_S)_3_ linker, which was not recognized by the G_4_S mAbs. It is conceivable that either this short motif can not be recognized by the antibody in general or that it may not be accessible due to protein folding. 

Compared to ligand-based assays (Miltenyi), the use of biotinylated anti-CAR linker antibodies resulted in a similar CAR T-cell detection rate for Axi-cel, Brexu-cel, and Liso-cel, respectively. Both, Ide-cel and Tisa-cel CARs show a distinctly lower sensitivity if detected by anti-CAR linker antibodies. It might be that the CD8a hinge domain, exclusively present in both CARs, may decrease antibody accessibility to the linker by altering scFv conformation. 

In general, an indirect CAR detection method using biotinylated antibodies is more sensitive, as it allows the binding of multiple fluorochrome conjugated anti-biotin Abs, which results in signal amplification.

Our data clearly shows that the specificity of anti-CAR linker antibodies is unaffected by the simultaneous presence of BiTEs/bsAbs targeting the same antigen as the CAR (Axi-cel, Liso-cel, Brexu-cel/blinatumomab, and Ide-cel/teclistamab). These results conclusively demonstrate diagnostic superiority compared to the standard ligand-based detection assays by allowing clinical CAR monitoring in most combination therapies. As a limitation, it should be noted that Tisa-cel CAR (G_4_S)_3_ linkers are not specifically detectable with anti-G_4_S linker mAb in combination with blinatumomab, as this synthetic BiTE even contains a (G_4_S)_3_ linker in its scFv [27]. The applicability of anti-CAR linker mAbs is also limited in cases where a patient has previously been treated with other antibodies that also incorporate the linker sequence (e.g., Glofitamab; CD20×CD3, containing a G_4_S linker).

In conclusion, the application of an anti-CAR linker mAb provides the following benefits: highly specific CAR detection, the recognition of a broad range of CARs, easy incorporation into multiparametric flow panels, and a considerably lower assay cost compared to that of ligand-based CAR detection assays by identical workload. In contrast to classical ligand-based CAR detection, it is applicable to the blood analysis of patients simultaneously treated with both BiTEs/bsAbs and CAR T-cells, targeting the same antigen with just one exception. Nevertheless, we also identified the following restrictions for the application of anti-CAR linker mAb: CARs containing a monomeric G_4_S linker are not recognized to be anti-G_4_S linker mAbs, the specificity of CAR detection is influenced by BiTEs containing the same linker as the CAR, and in some cases the sensitivity (percentage of positive cells) is distinctly lower compared to that of ligand-based CAR detection assays. 

In view of the increasing number of bispecific antibodies and CAR T-cell products being applied [28,29], anti-CAR linker antibodies are a new valuable tool to detect CAR expression.

## Data Availability

The original contributions presented in the study are included in the article/Supplementary Material, further inquiries can be directed to the corresponding author.

## Supplementary Materials

The following supporting information can be downloaded at: https://www.mdpi.com/article/10.3390/biomedicines12081641/s1; Figure S1: CAR detection with Alexa-conjugated anti-G_4_S mAb leads to false-positive results in whole blood specimens, if red blood cells are lysed by BD FACS™ Lysing solution; Figure S2: CAR T-cell detection by anti-F(ab) fragment antibodies leads to false-positive results in cases when bsAbs recognizes the same antigen as the CAR.
